# CYR61/CCN1 and WISP3/CCN6 are chemoattractive ligands for human multipotent mesenchymal stroma cells

**DOI:** 10.1186/1471-2121-8-45

**Published:** 2007-10-31

**Authors:** Norbert Schütze, Rita Schenk, Jörg Fiedler, Thomas Mattes, Franz Jakob, Rolf E Brenner

**Affiliations:** 1 Orthopedic Department, Orthopedic Center for Musculoskeletal Research, University of Würzburg, Würzburg, Germany; 2 Orthopedic Department, Division for Biochemistry of Joint and Connective Tissue Diseases, University of Ulm, Oberer Eselsberg 45, 89081 Ulm, Germany; 3 Orthopedic Department, University of Ulm, Ulm, Germany

## Abstract

**Background:**

CCN-proteins are known to be involved in development, homeostasis and repair of mesenchymal tissues. Since these processes implicate recruitment of cells with the potential to be committed to various phenotypes, we studied the effect of CYR61/CCN1 and WISP3/CCN6 on migration of human bone marrow derived mesenchymal stroma cells (MSCs) in comparison to in vitro osteogenic differentiated MSCs using a modified Boyden chamber assay.

**Results:**

CYR61 and WISP3 were purified as fusion proteins with a C-terminal Fc-tag from baculovirus infected SF21 cells using protein G sepharose columns. CYR61 and WISP3 stimulated cell migration of undifferentiated MSCs in a dose-dependent manner. CYR61 and WISP3 had similar effects on committed osteogenic precursor cells. Checkerboard analysis revealed that CYR61 and WISP3 stimulated true directed cell migration (chemotaxis) of MSCs and committed osteogenic precursors. In MSCs the chemotactic activity of WISP3 but not CYR61 was mediated through integrin *ανß*5.

**Conclusion:**

Our results indicate that CYR61 and WISP3 can function as soluble ligands transmitting chemotactic signals to human MSCs but differ in the involvement of integrin *ανß*5. This may be relevant for their possible role in connective tissue repair.

## Background

Members of the cysteine-rich 61/connective tissue growth factor/nephroblastoma overexpressed (CCN1-3) family of genes (CCN-family) have been reported to be involved in central cell-biological processes such as cell adhesion, migration, proliferation, differentiation and survival [1-3]. Additional CCN proteins are the wnt induced secreted proteins WISP1/CCN4, WISP2/CCN5 and WISP3/CCN6. The CCN proteins share a common modular structure comprising an insulin-like growth factor binding motif, a von Willebrand type C domain and a thrombospondin type I domain [3]. Based on the identical position of conserved cysteine residues within these proteins they are considered a structural family, but vary in function. CYR61 is considered as an angiogenic inducer, which binds to integrin receptors and proteoglycans [2,3].

Various CCN-proteins have been implicated in chondrogenesis and bone formation as well as angiogenesis during development and adult life [2]. These biological processes are also essential for skeletal tissue regeneration involving both endothelial and mesenchymal progenitor cells. Local recruitment of cells with the capacity to differentiate along the osteoblastic lineage for example is necessary for bone growth, remodeling and repair. The underlying cell-biological processes involve multipotent mesenchymal progenitor cells, which have been identified in bone marrow and various skeletal tissues including bone and cartilage [4-6]. Therefore, mechanisms regulating directed migration of undifferentiated cells might play an important role in skeletal physiology and pathology. Different chemokine receptors including CXCR4, CCR7, CCR1 and CX3CR1 have been identified on MSCs and stromal-derived factor (SDF)-1 has been shown to possess chemotactic activitiy for CXCR4-positive MSCs [7-9]. Further candidates for mediating chemotactic signals to bone marrow derived MSCs are growth factors, which are released during tissue remodeling or fracture healing. Thus it is not surprising that a chemotactic response of human bone marrow derived mesenchymal progenitor cells to factors like bone morphogenetic protein-2 (BMP-2), platelet-derived growth factor-isoforms (PDGF-AA, AB, BB), basic fibroblast growth factor (bFGF) or IGF-I and -II, has been demonstrated [10-14]. Some of these factors exhibited preferential or exclusive activity in osteoblastic cells while others showed enhanced activity in MSCs [13,15]. Vascular endothelial growth factor-A (VEGF-A), which has been shown to act as a coupling factor for angiogenesis and bone formation, stimulated directed cell migration of endothelial cells mainly via VEGFR2 and that of MSCs via VEGFR1 [15,16]. A dual function on bone formation and vascularisation has also been attributed to CYR61/CCN1 [17]. Recently, overexpression of CYR61/CCN1 has been identified as a chemotactic stimulus for the mesenchymal stem cell line C3H10T1/2 [18].

The observation that mutations in WISP3/CCN6 lead to progressive pseudorheumatoid dysplasia (PPRD) and polyarticular juvenile idiopathic arthritis, diseases that are associated with cartilage growth plate defects and/or loss of adult articular cartilage indicates that this CCN-protein might be related to the preservation of cartilage integrity [19,20]. It has been shown that WISP3/CCN6 stimulates collagen type II, aggrecan as well as superoxide dismutase expression and activity in chondrocytes [21,22]. Potential effects on mesenchymal stem cells, however, have not been described so far, although a differential expression of CCN-family members in human MSCs during osteogenic, chondrogenic or adipogenic differentiation has been reported. Both CYR61 and WISP3 were found to be expressed in undifferentiated MSCs. CYR61 expression generally seized with any differentiation along different lineages while WISP3 expression seized in chondrogenic differentiation, indicating that both polypeptides play important roles early in commitment or even in uncommitted MSCs [23].

The aim of this study was to establish recombinant expression of WISP3/CCN6 and to quantify possible chemotactic effects on primary human MSCs in comparison to CYR61/CCN1. Our results indicate that both CCN-proteins are chemoattractive ligands for human mesenchymal stroma cells before and after osteogenic commitment and that the effect of WISP3/CCN6 but not CYR61/CCN1 on cell migration of MSCs is mediated by the integrin receptor *ανß*5.

## Results

**Figure 1:**
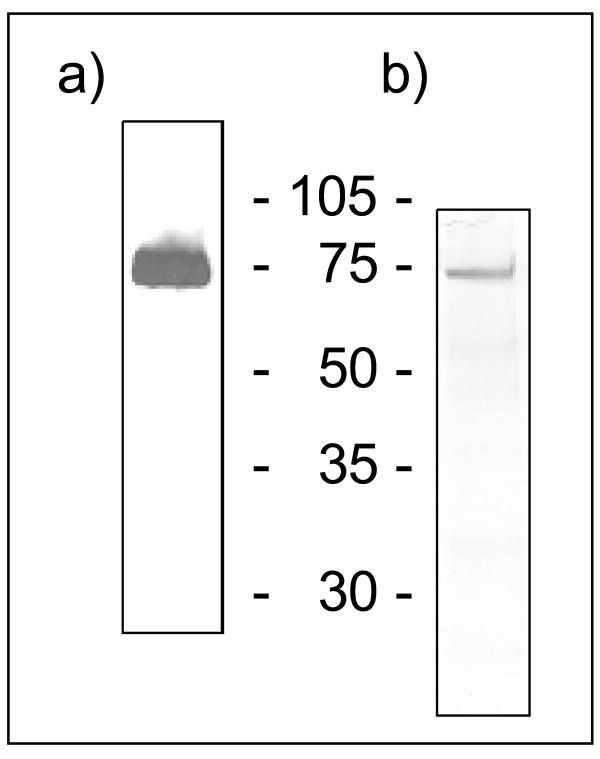
**Silver staining and western blot of recombinant WISP3-Fc**. To check the purity of WISP3-Fc SDS gel electrophoresis followed by silver staining and western blotting were applied as described in materials and methods. a) western-blotting analysis, b) silver stained SDS gel. Size of the fusion protein is 72 kDa.

Silver staining and western blotting revealed the purity of the recombinant WISP3-Fc protein (Figure 1). A major band of the expected size (72 kDa) was visible in these analyses. From two 150 cm^2 ^flasks the purification subsequently yielded 100 to 250 μg protein.

Human MSCs used for the chemotaxis assays did not express markers of differentiated osteoblasts (osteocalcin), chondrocytes (collagen type II) or adipocytes (leptin). Immunostaining of MSCs showed that *ανß*5 integrin was expressed on the protein level while *ανß*3 integrin could scarcely be detected (data not shown). Addition of dexamethasone, ascorbic acid and ß-glycerophosphate led to osteogenic differentiation within 21 days, indicated by the expression of alkaline phosphatase and osteocalcin (data not shown).

**Figure 2:**
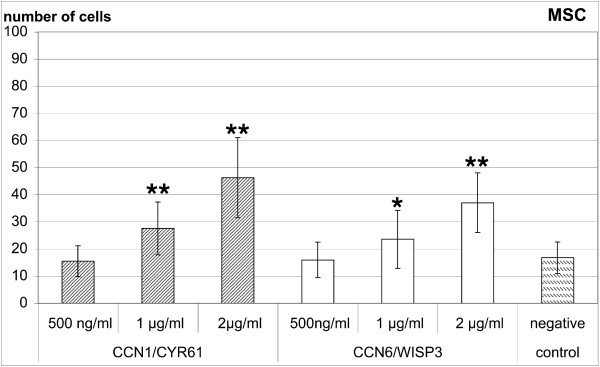
**CYR61 and WISP3 stimulate migration of human MSCs**. CYR61/CCN1 and WISP3/CCN6 stimulate migration of human MSCs in a dose dependent manner. Results are presented as absolute numbers of migrated cells from six independent experiments, measured in quadruplicate each (mean ± SD, * p < 0.05, ** p < 0.01 versus negative control in paired t-test).

**Figure 3:**
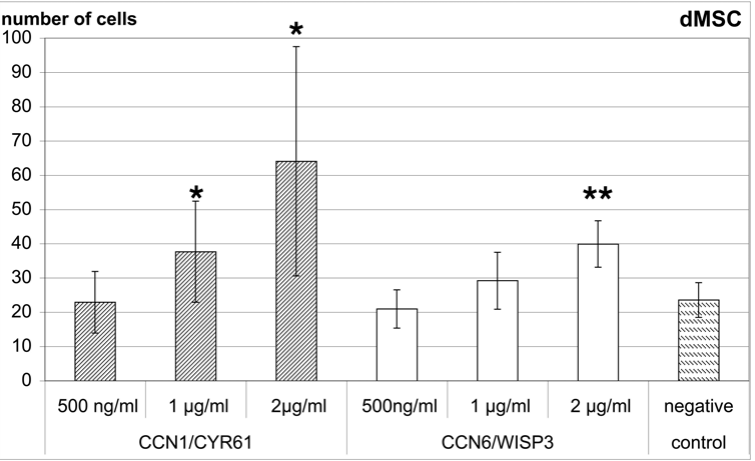
**CYR61 and WISP3 stimulate migration of osteogenic differentiated MSCs**. CYR61/CCN1 and WISP3/CCN6 stimulate migration of osteogenic differentiated human MSCs in a dose dependent manner. Results are presented as absolute numbers of migrated cells from six independent experiments, measured in quadruplicate each (mean ± SD, * p < 0.05, ** p < 0.01 versus negative control in paired t-test).

Full length CYR61 and WISP3 induced a dose-dependent migratory response in human bone marrow derived MSCs through gelatin-coated chemotaxis filters (Figure 2). An effect could be observed starting with 1 μg/ml of both recombinant proteins. With 2 μg/ml migration of 46.3 ± 14.8 cells per well could be observed for CYR61 and 37.0 ± 11.0 cells per well for WISP3 in comparison to 16.8 ± 5.8 cells per well for control medium (Figure 2). For MSCs stimulation with both CCN-proteins was significant at 1 and 2 μg/ml. Single experiments indicated that a further increase in the concentration of both CCN-proteins to 5 μg/ml and 10 μg/ml result in a gradual decrease of the migratory response (data not shown). The number of migrated MSCs compared to 100 ng/ml rhIGF-I was 81% for 2 μg/ml CYR61 and 74% for 2 μg/ml WISP3. BSA as a negative control had no stimulatory effect at 2 μg/ml (26.6 ± 3.5 versus 30.1 ± 1.6 of control medium, M ± SD, n= 3). For in vitro differentiated MSCs the effect of CYR61 and WISP3 was in a comparable range although the variation with CYR61 was higher (Figure 3). For dMSCs the effect of CYR61 was significant at 1 and 2μg/ml and that of WISP3 at 2 μg/ml.

**Figure 4:**
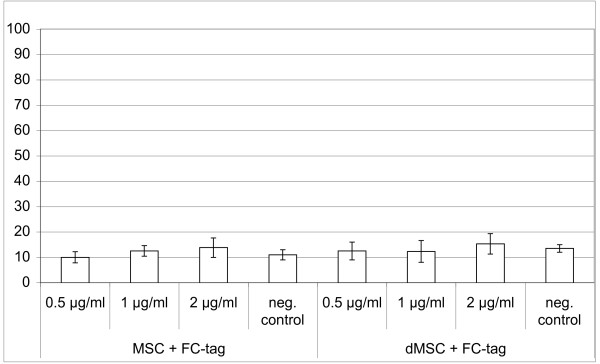
**The Fc-tag does not influence migration**. The Fc-tag has no influence on migration of human MSCs and osteogenic differentiated MSCs up to 2 μg/ml. Results are presented as absolute numbers of migrated cells measured in quadruplicate (mean ± SD) from one representative experiment out of two independent experiments.

Since the recombinant proteins both contained the IgG Fc-tag it was necessary to test whether the Fc-tag itself influenced cell migration of MSCs or dMSCs. As shown in Figure 4 we could not observe any relevant stimulation of cell migration above basal values in concentrations of 0.5, 1 and 2 μg/ml indicating that the migratory effect of the recombinant protein constructs is specific due to the function of CYR61 or WISP3.

To rule out the possibility that CYR61 and WISP3 induced chemokinesis, and not directed cell migration, a checkerboard analysis was performed with the most effective concentration of 2 μg/ml. These results (Table 1) clearly demonstrated that both CCN-proteins stimulated migration of human MSCs and dMSCs only at a positive concentration gradient.

**Table 1:** Checkerboard analysis for migratory effects of CYR61/CCN1 and WISP3/CCN6.

	Well	MSC	dMSC
negative control		14.3 ± 2.9	16.3 ± 3.7
CCN1/CYR61	lower	55.5 ± 6.5	40.3 ± 4.0
	upper&lower	18.8 ± 4.4	15.0 ± 2.9
CCN6/WISP3	lower	43.5 ± 3.2	30.8 ± 3.3
	upper&lower	12.0 ± 2.2	13.5 ± 4.2

Checkerboard analysis for migratory effects of CYR61/CCN1 and WISP3/CCN6 with human MSCs and in vitro osteogenic differentiated MSCs (dMSC). Results are presented as absolute migrated cells (mean ± SD of one quadruplicate determination). 2 μg/ml CYR61/CCN1 or 2 μg/ml WISP3/CCN6 were added only to the lower compartment (lower) or to both the upper and lower compartment (upper&lower) of the chemotaxis chamber to eliminate the concentration gradient.

**Figure 5:**
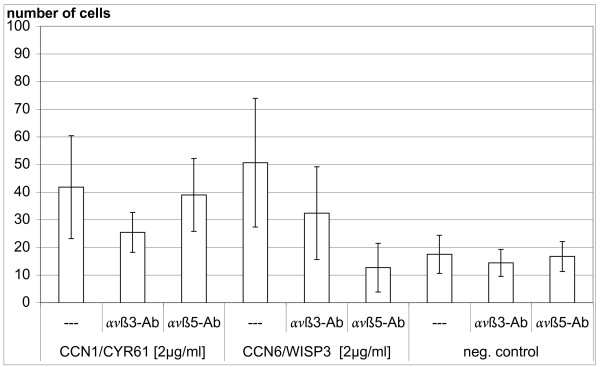
**Integrin *ανß*5 mediates MSC migration to WISP3 but not to CYR61**. Integrin *ανß*5 mediates MSC migration to WISP3/CCN6 but not to CYR61/CCN1-protein. Results are presented as absolute numbers of migrated cells from three independent experiments measured in quadruplicate each (mean ± SD). MSCs were partly preincubated for 1 h with 50 μg/ml *ανß*3 (LM609) or *ανß*5 (P1F6) antibody (Ab) as indicated. CYR61 and WISP3 were used at a concentration of 2 μg/ml each.

**Figure 6:**
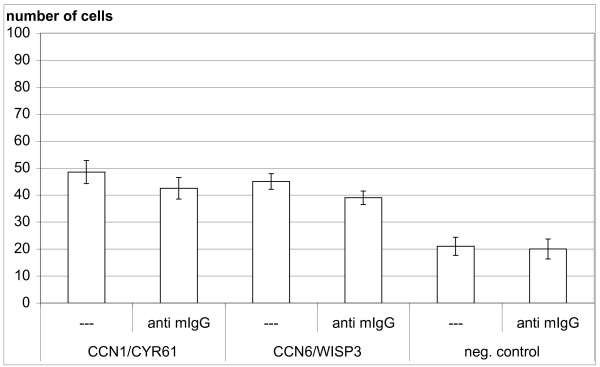
**Normal mouse IgG does not influence MSC migration to CYR61 or WISP3**. Preincubation with normal mouse IgG does not influence the response of MSCs to 2 μg/ml CYR61 or 2 μg/ml WISP3. Results are presented as absolute numbers of migrated cells measured in quadruplicate (mean ± SD) from one representative experiment out of two independent experiments.

Preincubation of MSCs with the integrin *ανß*3 antibody did not have a strong effect on the migratory response to CYR61 and WISP3 protein. Preincubation with the integrin *ανß*5 antibody did not affect CYR61 induced migration but completely prevented the effect of WISP3 (Figure 5). In contrast, preincubation with normal mouse IgG at the same concentration (50 μg/ml) did not influence the migratory response to both CCN-proteins (Figure 6).

## Discussion

A chemotactic effect of CYR61/CCN1 has been previously described for fibroblasts, alpha smooth muscle cells, endothelial cells and the murine mesenchymal stem cell line C3H10T1/2 [18]. We could extend this spectrum to the stimulation of cell migration in human primary MSCs before and after osteogenic differentiation. This is of special importance since in recent years it has turned out that locally available, bone-marrow-derived mesenchymal or osteo-progenitor cells are crucially involved in processes such as bone growth, remodeling or repair [24]. Therefore, the mechanisms regulating migratory signals are of central importance for our understanding of the physiology and pathology of bone tissue.

This is the first report that beside full-length CYR61/CCN1 also full length WISP3/CCN6 acts as a soluble factor stimulating migration of human bone-marrow-derived MSCs and dMSCs in a dose-dependent manner. Moreover, the stimulation could be specified as chemotaxis and not chemokinesis and, thus, may contribute to site-directed cell recruitment in vivo. These results confirm the biological activity of both recombinant expressed proteins. 2 μg/ml of the recombinant CCN-proteins in which the Fc-tag accounts for nearly half of the protein lead to about 70–80% of migrated cells compared to 100 ng/ml rhIGF-I. The concentrations needed for a similar chemotactic effect of these matricellular proteins were therefore markedly higher than for other growth factors but they were comparable to concentrations of CCN-proteins known to elicit cell-biologic responses in previous studies. The characteristics as matricellular proteins in tightly binding to heparan sulfate proteoglycans and distinct integrin receptors as well as the solubility of these proteins may be related to this point. For chemotactic processes along far distances proteolytic fragments of these domain structured proteins might be involved, which remains to be tested by functional domain bashing analysis in future experiments. In this context it might be relevant that IGFBP5 has a direct chemotactic effect on human MSCs [14]. A similar functional relevance of the IGFBP-domain of CCN proteins, however, has not been shown so far.

We have previously shown that in vitro differentiation of MSCs to the osteogenic phenotype by dexamethasone, ascorbic acid and ß-glycerophosphate mostly led to a chemotactic response very similar to that of primary osteoblasts [12]. For the CCN-proteins CYR61 and WISP3 we did not find significant differences between uncommitted and osteogenic committed MSCs. The aspect of lineage-specific effects, however, deserves more detailed investigation [23]. Integrin receptor interaction has previously been recognized as an important factor with cell-type- and function-specific influence [25-27]. In agreement with such an influence, a relevant stimulation of cell migration in response to CYR61/CCN1 and WISP3/CCN6 could not be observed with native but with gelatin-coated polycarbonate filters which have been used in most previous studies on migratory effects of CCN-proteins [25,27-30]. Chemotactic effects of CYR61 are known to be mediated by integrin *ανß*3 in endothelial cells and by integrin *ανß*5 in fibroblasts [25,31]. Our results indicate that in MSCs *ανß*3 integrin does not strongly contribute to the transmission of migratory signals by CYR61 and WISP3-proteins. Most interestingly, we found that *ανß*5 integrin is not involved in MSC migration to CYR61 while the chemotactic effect of WISP3 was completely dependent on this integrin receptor. In agreement with these results immunostaining indicated that *ανß*5 integrin was expressed on MSCs while *ανß*3 integrin could scarcely been detected. These results represent the first data on the role of integrin receptors in WISP3 induced cell migration and indicate ligand-specific involvement of integrin receptors within CCN-proteins. A possible role of other integrin receptors like α6ß1 or heparan sulfate proteoglycans for CYR61 and WISP3 mediated stimulation of MSC-migration has to be addressed in future studies. Moreover, the relationship to chemoattractive effects of certain chemokines and other growth factors on MSCs is largely unknown so far.

## Conclusion

Our in vitro data show that recombinant CYR61 and WISP3-proteins both stimulate directed migration of human MSCs and committed osteogenic precursor cells. The chemotactic activity of these two members of the CCN-protein family differently involves integrin *ανß*5 in MSCs, indicating a further level of regulation. This may be relevant for MSC recruitment and migration in the context of skeletal development and repair.

## Methods

### Cell Culture

With informed consent, following the guidelines from the ethics committee of the University of Ulm, bone marrow samples from eight donors (age 20 to 39 years) were plated and cultured in Dulbecco's modified eagle medium (DMEM; Biochrom, Germany) containing 10% FCS as described previously [12]. All cell culture experiments were performed in the first four cell passages. The technique of cell isolation and cultivation preserves the multipotency phenotype, indicated by absent or very low expression of alkaline phosphatase or osteocalcin, and the potential to differentiate to the chondrogenic, osteogenic and adipogenic phenotype [32]. The cell populations were characterized by positive staining with anti-CD9, CD54, CD166, and STRO-1 as cell surface markers known to be expressed by human MSCs [32]. Immunostaining for integrin *ανß*3 and integrin *ανß*5 was performed with receptor specific antibodies (LM609 for *ανß*3 and P1F6 for *ανß*5, both Chemicon International, United Kingdom) using hemalaun as nuclear counterstain to improve visualization of the cells. SF-21 insect cells were cultured in BacPAK complete medium (Clontech, Saint-Germain-en-Laye France) at 27°C. SF-21 cells were split and medium was changed once a week.

### Osteogenic differentiation of MSCs and analysis of gene expression

Medium for osteogenic differentiation, containing DMEM with 0.1 μM dexamethasone, 10 mM ß-glycerophosphate and 50 μg/ml ascorbic acid, was changed every third day as described [12]. Osteogenic differentiation was demonstrated at day 21 by RT-PCR gene expression analysis of the marker genes alkaline phosphatase and osteocalcin as described elsewhere [16].

### Cloning for WISP3 expression

The open reading frame for WISP3 was amplified from a plasmid (IMAGp956O19142) obtained from the Resource Center Primary Database (Berlin, Germany). Primers corresponding to the translational start site (including the KOZAK Sequence of the WISP3 cDNA sequence) and the stop site (the primer lacked the stop codon) contained restriction sites at the 5'end (*Xho*I and *Eco*RI in the forward and reverse primer, respectively). The PCR product was subcloned into a TA-TOPO vector (pCR 2.1, Invitrogen, Karlsruhe, Germany). The insert was prepared by double restriction (*Eco*RI/*Xho*I) and cloned into the transfer vector pBakPak 8 (Clontech, Saint-Germain-en-Laye France). The Fc-domain of human IgG was amplified from a PC3 expression vector (kindly provided by Dr. Pascal Schneider, University of Lausanne, Switzerland) by PCR using primers with EcoRI and NotI restriction sites, respectively and was subcloned 3' to the open reading frame of WISP3 to generate the final recombinant vector.

### Transfection of insect cells and expression of full-length CYR61 and WISP3 proteins

SF-21 caterpillar (lepidopteran) derived cells were used to express recombinant WISP3-protein (rWISP3). The recombinant vector was mixed with linearized DNA that contained a modified *Autographa californica *multiple nucleopolyhedrovirus (AcMNPV) genome. SF-21 cells were transfected with bacfectin according to the supplier's information in wells of 6-well plates. After 7 days at 27°C in serum containing medium the supernatant (passage 1) was used to infect SF-21 cells in 25 cm^2 ^flasks using serum-free culture conditions. After 7 days the supernatants (passage 2) were checked for protein expression and further amplified one round using 75 cm^2 ^flasks to generate the final stock (passage 3) which was stored in aliquots at -80°C for further purifications of the protein. To purify the rWISP3-protein, protein G sepharose columns (1 ml columns, GE Healthcare, Freiburg, Germany) were equilibrated with pH 7 buffered PBS, the supernatant was applied (volumes between 45–90 ml) at a flow rate of 1 ml per min. Columns were washed with 10 column volumes of PBS and the protein eluted with elution buffer (0.1 M glycine, pH 2.2). Directly thereafter eluted fractions were neutralized using 3 M Tris/HCL pH 8. The yield was determined by a Bradford protein assay. The purity of the protein was finally checked by SDS gel electrophoresis followed by silver staining and western blotting. CYR61 was purified as described previously [17].

The purification of the Fc-tag was done in parallel using similar procedures. The Fc-domain of human IgG was amplified from a PC3 expression vector (kindly provided by Dr. Pascal Schneider, University of Lausanne, Switzerland) by PCR using primers containing EcoRI and NotI restriction sites and subcloned into the transfer vector pBakPak 8 (Clontech, Saint-Germain-en-Laye France). Further virus amplification and protein purification was done essentially as described above for the CCN-proteins.

### Chemotaxis Assay

Cell migration was analyzed by a modified Boyden chamber assay, using a 48-well micro-chemotaxis chamber (NeuroProbe Inc., Baltimore, MD, USA) and polycarbonate filters with 8 μm pores (Whatman Biometra, Göttingen, Germany), as described previously [14]. For the present study the filters were coated with gelatin. The cells were trypsinized, counted and used for RT-PCR as well as for the chemotaxis assay. The lower wells were filled with 0.5, 1 and 2 μg/ml of the CCN-proteins in DMEM and covered by the chemotaxis filter. The protein concentration was routinely tested using a Bradford assay. The upper wells were filled with 1 × 10^4 ^cells in 50 μL DMEM. After 4 h incubation, the filter was carefully removed and non-migrated cells on the upper side eliminated by rinsing with cold PBS and scraping over a rubber wiper. The migrated cells in the solution on the lower side of the filter were fixed with 4% formaldehyde and stained with "Giemsa". Control wells only containing DMEM in the bottom well were applied for each experiment. Conditioned medium of human osteoblast cultures was used as a positive control, analogous to the use of conditioned medium from fetal cultures for fibroblast chemotaxis [33]. In control wells, containing only DMEM, the number of migrated cells was about 10 to 30 cells, while the amount of migrated cells in the wells with conditioned medium was greater than 1000 cells. Additional control experiments were performed with the Fc-tag in concentrations of 0.5, 1 and 2 μg/ml using MSCs and dMSCs from two different donors. As a negative control the effect of 2 μg/ml BSA (Sigma, Deisenhofen, Germany) was tested with MSCs from three different donors. In a positive control experiment the effect of 100 ng/ml rhIGF-I (Pepro Tech Inc, Rocky Hill, NJ, USA) on MSC-migration was tested in parallel to CYR61 and WISP3. The number of migrated cells in control and stimulated wells was counted at 100-x magnification. A Zigmond-Hirsch checkerboard analysis was performed in triplicate to distinguish between concentration-dependent cell migration (chemotaxis) and random migration (chemokinesis). Therefore, the effect of elimination of the concentration gradient by adding the test substances (2 μg/ml each) to the upper and lower compartments of the chemotaxis chamber [34] was tested in parallel. Finally, the involvement of integrin receptors *ανß*3 and *ανß*5 was studied by preincubation of MSCs with specific antibodies (LM609 for *ανß*3 and P1F6 for *ανß*5, Chemicon International, United Kingdom) for 1 h at a concentration of 50 μg/ml each. Preincubation with 50 μg/ml normal mouse IgG (Sigma, Deisenhofen, Germany) was used as an unspecific control.

### SDS gel electrophoresis, silver staining and western blotting

SDS gel electrophoresis, silver staining and western blotting essentially were performed as described previously [17]. The WISP3-protein was detected using a polyclonal antiserum raised in rabbits against the full-length human CYR61-Fc fusion protein, which detects the Fc-tag. Controls using the secondary antibody only were negative.

### Statistics

The chemotaxis experiments on dose-dependent effects of CYR61 and WISP3 were performed in quadruplicate, for MSCs and osteogenic differentiated MSCs (dMSCs) in independent experiments with cells from six different donors, and the mean value for each donor was used for statistical analysis. Experiments with integrin-blocking antibodies were done in quadruplicate determinations with cells from three different donors each. Results are presented as mean ± standard deviation. The significance of differences in chemotactic responses compared to the negative control was determined using Student's *t*-test for paired samples. A value of *P *<0.05 was considered statistically significant.

## Authors' contributions

NS synthesized recombinant CYR61 and WISP3 and participated in drafting the manuscript, RS established recombinant synthesis of CYR61 and WISP3, JF was involved in the experimental design of chemotaxis experiments and data evaluation, TM aspired bone marrow samples and contributed to the establishment of MSC cultures, FJ participated in data interpretation and drafting of the manuscript, RB designed the study and drafted the manuscript. All authors read and approved the final manuscript.
